# Sources and Distribution of Surface Water Fecal Contamination and Prevalence of Schistosomiasis in a Brazilian Village

**DOI:** 10.1371/journal.pntd.0003186

**Published:** 2014-10-02

**Authors:** Rafael Ponce-Terashima, Amber M. Koskey, Mitermayer G. Reis, Sandra L. McLellan, Ronald E. Blanton

**Affiliations:** 1 Mercer University School of Medicine, Macon, Georgia, United States of America; 2 Center for Global Health and Diseases, Case Western Reserve University, Cleveland, Ohio, United States of America; 3 School of Freshwater Sciences, Great Lakes Water Institute, University of Wisconsin, Milwaukee, Wisconsin, United States of America; 4 Laboratory of Pathology and Molecular Biology, Gonçalo Moniz Research Center, Oswaldo Cruz Foundation, Salvador, Bahia, Brazil; University of Florida, United States of America

## Abstract

**Background:**

The relationship between poor sanitation and the parasitic infection schistosomiasis is well-known, but still rarely investigated directly and quantitatively. In a Brazilian village we correlated the spatial concentration of human fecal contamination of its main river and the prevalence of schistosomiasis.

**Methods:**

We validated three bacterial markers of contamination in this population by high throughput sequencing of the 16S rRNA gene and qPCR of feces from local residents. The qPCR of genetic markers from the 16S rRNA gene of *Bacteroides*-*Prevotella* group, *Bacteroides* HF8 cluster, and *Lachnospiraceae* Lachno2 cluster as well as sequencing was performed on georeferenced samples of river water. Ninety-six percent of residents were examined for schistosomiasis.

**Findings:**

Sequence of 16S rRNA DNA from stool samples validated the relative human specificity of the HF8 and Lachno 2 fecal indicators compared to animals. The concentration of fecal contamination increased markedly along the river as it passed an increasing proportion of the population on its way downstream as did the sequence reads from bacterial families associated with human feces. *Lachnospiraceae* provided the most robust signal of human fecal contamination. The prevalence of schistosomiasis likewise increased downstream. Using a linear regression model, a significant correlation was demonstrated between the prevalence of *S. mansoni* infection and local concentration of human fecal contamination based on the *Lachnospiraceae* Lachno2 cluster (r^2^ 0.53) as compared to the correlation with the general fecal marker *E. coli* (r^2^ 0.28).

**Interpretation:**

Fecal contamination in rivers has a downstream cumulative effect. The transmission of schistosomiasis correlates with very local factors probably resulting from the distribution of human fecal contamination, the limited movement of snails, and the frequency of water contact near the home. In endemic regions, the combined use of human associated bacterial markers and GIS analysis can quantitatively identify areas with risk for schistosomiasis as well as assess the efficacy of sanitation and environmental interventions for prevention.

## Author Summary

People tend to live close to natural water bodies, and often these water bodies are used as waste disposal in many regions of the world. The consequences of this are often studied with regard to bacterial and viral infections, but rarely for parasitic infections. In this study the authors examined a rural community settled along a river in Brazil, and found that the concentration of fecal bacteria in water accumulates as the river runs downstream. Molecular methods were able to show that most of these fecal bacteria were of human origin rather than from local livestock or other domestic animals. To assess the impact of fecal contamination of surface waters, the authors investigated its association with schistosomiasis, a parasitic infection transmitted by snails exposed to water contaminated by human feces. Similar to the distribution of fecal contamination, the proportion of people with schistosomiasis was higher in areas located downstream. A model combining concentration of human fecal bacteria in water and Geographic Information Systems (GIS) analysis of schistosomiasis prevalence showed that areas of increased concentration of human feces correlated with areas in the village at higher risk for schistosomiasis. This research provides insight into the dynamics of fecal contamination of rivers and its spatial impact on a human parasitic disease.

## Introduction

The culture of common fecal organisms such as coliforms and enterococci from surface waters has historically been used as a proxy for the risk of infection with viral, bacterial, and parasitic pathogens [1]. It forms the standard for the United States Environmental Protection Agency's criteria for water quality [2]. Despite the well-studied association between fecal contamination of water and acute enteric and skin diseases [2], [3], a correlation between these bacterial proxies and specific disease causing organisms has been difficult to demonstrate in the absence of a point-source such as sewage outflows [4]. Known limitations that could explain this weak association include the short survival of some fecal indicator organisms in water [5], their presence in environmental sources including soils and sediments [6], [7], contributions from non-human sources, and low sensitivity of detection methods for some pathogens [6], [7]. The short incubation and shedding periods of these infections may also cause the pathogenic organism to no longer be present in sampled water by the time an investigation is undertaken. Molecular methods have been developed to address some of these weaknesses. Most current approaches involve PCR amplification of bacterial rDNA taken directly from specific hosts or sources of fecal contamination without prior culture. Fecal anaerobic bacteria are some of the most promising alternative indicators to *Escherichia coli* and enterococci. They are more abundant than coliforms, they do not multiply in the water column, and some sub-species or strains are more specific for host sources [8]–[12]. One of the most commonly employed and reliable indicators for human fecal pollution are human Bacteroides originally described as the HF8 cluster [13], [14]. A concern remains as to the distribution of these markers originally developed in the US and other developed countries and whether they can be associated with disease risk in other parts of the world [15].

In contrast to most waterborne bacterial and viral infections, schistosomiasis is a chronic parasitic infection that results from skin contact with water as opposed to ingestion. It is a global disease that is transmitted in 78 countries with 240 million people infected [16]. In Brazil, where it is the second most common cause of morbidity and death due to parasitic infection [17], *Schistosoma mansoni* is the only human species transmitted. Its transmission in common with other waterborne diseases is dependent on human fecal contamination of fresh water. Thus, in Brazil at a national scale the distribution of schistosomiasis maps to areas with the poorest level of sanitation [18].The parasite is able to establish a long-term infection (5–40 years) that produces hundreds to thousands of eggs per day, most of which will be eliminated in the feces [19]. An obligate step in transmission is development in a host snail, so that there can be no direct temporal connection between fecal contamination and human infection. Snails movements, however, are extremely restricted geographically [20] and in this way past contamination and infection events are registered locally in chronic human infections.

Given the complex life cycle of this parasite and its long-term survival in a community, bacterial indicators that track human sources of fecal contamination in water may contribute much to our understanding of the transmission dynamics of the parasite. Since snail infection with *S. mansoni* is dependent on human fecal contamination of surface waters, the probability of snail infection and differences in the spatial distribution of human schistosomiasis is likely to correlate with differences in the concentration and distribution of this contamination. We tested this in one small community in Northeastern Brazil.

## Materials and Methods

The study was conducted in a Brazilian village. Demographic and schistosomiasis prevalence data was obtained from a population and fecal survey whose results have been previously published [21], [22]. DNA was extracted from stool samples of individuals who tested positive for schistosomiasis. Animal fecal samples were also collected for DNA extraction. We validated use of three host-indicative fecal bacterial markers in this population by high throughput sequencing of 16S rRNA gene and qPCR of human and animal feces. Water samples were collected along the village's main river. Fecal water contamination was assessed by traditional culture as well as by qPCR of host-indicative fecal markers. Sequence data from the microbial communities found in river water was used to compare the relative abundance of >20 bacterial families. Finally, by use of a linear regression model, we tested the correlation of household proximity to concentration of human fecal water contamination with prevalence of schistosomiasis.

### Ethics statement

The Committee on Ethics in Research of the Oswaldo Cruz Foundation of Salvador, Bahia, the Brazilian National Committee on Ethics in Research, and the Institutional Review Board for Human Investigation of University Hospitals Case Medical Centre, Cleveland, Ohio approved the study design. Study participants provided written informed consent. Animal owners provided permission to collect the stool samples of their animals.

### Study site, population & spatial analysis

**Figure 1:**
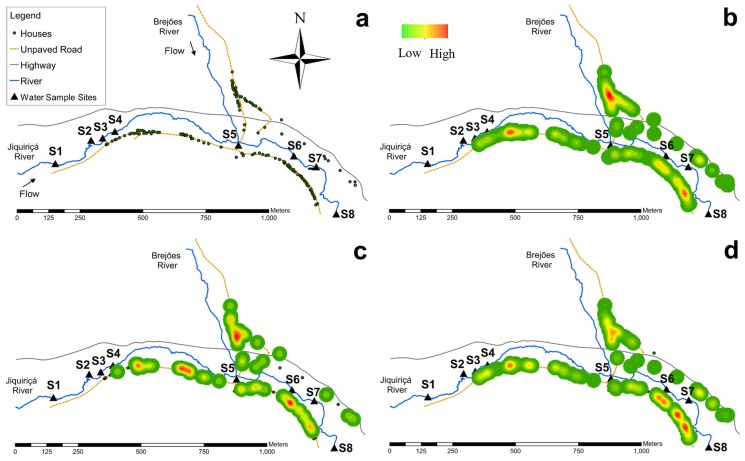
(a) Study area: village of Jenipapo in the state of Bahia, Brazil. (b) Kernel density distribution of human population. (c) Kernel density distribution of sewage draining directly into the river. (d) Kernel density distribution of *S. mansoni* infection.

The village of Jenipapo in the state of Bahia, Brazil was selected for study because of its high prevalence of *S. mansoni* infection, the geographic distribution of its human population around surface waters, and its relative isolation from other settlements. The village is split north and south by the Jiquiriçá River and a two-lane highway. The Brejões River descends from the north, borders part of the village on the west, and enters the Jiquiriçá River at approximately the village midpoint (Fig. 1a). Within Jenipapo, the Jiquiriçá River measures 5–10 meters across and less than 1 m deep, with areas of bare rock as well as thick aquatic vegetation. The Brejões is narrower and shallower, but still perennial. Most houses are located within 20 meters of these rivers. Topographically, the region is a narrow river valley with approximately equal elevations on both sides. Commercial activity is primarily devoted to raising livestock along with planting cassava, beans, and bananas. Demographic data and prevalence of schistosomiasis was obtained from interviews and a fecal survey of all residents of the village in 2009. The description of the community has been published previously [21], [22].

The location of each home and human water contact sites in the community was registered with a hand-held Trimble/Nomad GPS unit (Model 65220-11). The course of the river within the village was surveyed by walking along one bank. Data were imported into ESRI ArcGIS 10.0 (Redlands, CA) for mapping and analyses. Kernel density estimation was used to assess and display the spatial density of the human population, schistosome infection, and river use for sewage disposal. The Moran's I statistic was calculated using the Spatial Autocorrelation Tool in ArcGIS to assess spatial clustering.

### Schistosomiasis diagnostic assay

The collection and processing of human fecal samples of the residents of Jenipapo was described previously [22]. Briefly, all inhabitants of the community >1 year of age were asked to enroll in the study, and in addition to answering a questionnaire, they provided a stool on 3 different days. A single slide was prepared by the Kato-Katz method [23]. Microscopists trained for parasitological studies read one slide per sample, and from this the number of eggs per gram of stool (epg) in each sample was determined. Participants with one or more egg-positive stools were treated with praziquantel at dosages recommended by Brazilian Ministry of Health.

### Sample collection and DNA extraction

#### Human fecal samples

DNA was extracted from fecal samples of individuals who tested positive for schistosomiasis. As part of studies on parasite population genetics, whole stools positive for *S. mansoni* were liquefied and passed through a series of sieves that retained eggs and much of the fecal solids. The bottom 5 ml of sediment was collected and then kept frozen at −20°C until used for DNA isolation. The human fecal samples were extracted by standard phenol/chloroform protocol followed by further cleanup using hexadecyltrimethyl ammonium bromide (CTAB) to remove PCR inhibitors [24]. Individual stool samples were assigned sample numbers, and 10 samples were selected by random number generator for next generation sequencing.

#### Animal fecal samples

Fresh stools were collected from three pigs, three dogs, two cows, and two horses residing in Jenipapo. Approximately 10 g were collected from each with a plastic scoop and stored in 2 ml screw cap tubes at −20°C. Efforts were made to collect samples within minutes after deposition to limit changes in bacterial numbers and to prevent environmental contamination. DNA was extracted from 200 mg of the sample using a Qiagen Stool Kit (Qiagen, Valencia, CA) according to the manufacturer's protocol. Extracted DNA was stored at −20°C until further analysis.

#### Water samples

A total of eight water sample sites were chosen along the Jiquiriçá River. Six of the eight sites were most commonly reported for human contact with water (Fig. 1a). Of the remaining two, one was collected 10 meters upstream from the first house and the other 55 meters downstream of the last house. An additional sample was collected from a small pond >4 km from the village. This was the source of the community's drinking water located distant from human habitation. Water samples were collected in August of 2012 between 8A and 3P using clean, dry 500 ml plastic bottles. No more than trace rainfall had occurred in the region in the last week. Where possible, a sample was taken from both banks of the river. Each bottle was stored at 4°C within 30 min of collection. The sample was then filtered through a 0.22-µm nitrocellulose filter (EMD Millipore Corporation, Billerica, MA). Filters were folded and placed in 2 ml screw cap tubes and stored at −20°C for one week in the field and −80°C in the laboratory until DNA extraction. For DNA extraction, frozen filters were broken into small fragments with a sterile metal spatula and vortexed with a bead-beating matrix and buffers, according to the manufacturer's instructions for the Fast DNA SPIN Kit for Soil (MP Biomedicals, Solon, OH).

### Quantification of traditional and alternative indicators

One ml of each sample was placed in culture media (3M Petrifilm E. coli/Coliform Count Plate, 3M, Saint Paul, MN) for 24 h at 37°C and counted for colony forming units (CFUs) of total coliforms and *E. coli*.

Six different qPCR assays were used in extracted DNA of all collected water samples for identification and quantification of fecal bacteria indicative of human or ruminant sources (Table 1). All qPCR assays were amplified in 25 µl reactions using 12.5 µl TaqMan Master mix, 1.0 µl 25 µM primer mixtures, 1.0 µl 2 µM probe mixtures, 5.5 µl water and 5.0 µl of DNA. Assays were carried out as previously described in the referenced literature in Table 1. All assays were run in duplicate.

**Table 1:** Primers used in this study.

*Bacterial target*	Target Host	Primer	Sequence	Reference
*Bacteroides-Prevotella group*	Non specific	GenBac3F	GGGGTTCTGAGAGGAAGGT	[42]
		BacsppR	CCGTCATCCTTCACGCTACT	[42]
		Bacspp346p	[6FAM]-CAATATTCCTCACTGCTGCCTCCCGTA-[MGBNFQ]	[43]
*Bacteroides HF8 cluster*	Human indicative	HF183F	ATCATGAGTTCACATGTCCG	[13]
		BacHum241R	CGTTACCCCGCCTACTATCTAATG	[44]
		BacHum193p	[6-FAM]-TCCGGTAGACGATGGGGATGCGTT-[MGBNFQ]	[45]
*Lachnospiraceae Lacno2 cluster*	Human indicative	Lachno2F	TTCGCAAGAATGAAACTCAAAG	[46]
		Lachno2R	AAGGAAAGATCCGGTTAAGGATC	
		Lachno2p	[6FAM]-ACCAAGTCTTGACATCCG-[MGBNFQ]	
*E. coli*	Non specific	uidA1663F	GCGACCTCGCAAGGCATA	[47]
		uidA1790R	GATTCATTGTTTGCCTCCCTGCTGCG	
		uid1729p	[6FAM]-TGCAGCAGAAAAGCCGCCGACTTCGG-[MGBNFQ]	
*Bacteroidetes*	Ruminant indicative	BacR_f	GCGTATCCAACCTTCCCG	[48]
		BacR_r	CATCCCCATCCGTTACCG	
		BacR_p	[6FAM]-CTTCCGAAAGGGAGATT-[MGBNFQ]	

### Microbial community analysis Next Generation Sequencing

Deep sequencing using the Illumina MiSeq platform was carried out at the Josephine Bay Paul Center of the Marine Biological Laboratory. A comprehensive microbial community profile was generated for five river samples, ten human fecal samples, and all collected animal fecal samples. The V6 hypervariable regions of the 16S rRNA gene were amplified in each of the samples using previously described primers and protocols [25]. Sequences were trimmed, controlled for low quality and contaminated reads, and then aligned. Nearly 27 million bacterial sequence reads were generated (∼1 million reads per sample). The sequence data were further processed and stored in the Visualization and Analysis of Microbial Population Structures (VAMPS) database (http://vamps.mbl.edu) [26]. Taxonomic assignments were made for all sequences using Global Alignment for Sequence Taxonomy (GAST) [27]. Further analysis of sequence data is reported in [28] and sequence data is available in the National Center for Biotechnology Information (NCBI) Short Read Archive under the accession number SRP041262.

To assess the proportion of bacterial community members that are potentially amplified by the human-indicative fecal indicator assays, a BLAST search was performed against the Illumina sequence data sets with the HF8 and Lachno2 primers [29]. Since the primers for the human-indicative assays are in regions of the 16S rRNA gene different from or only encompasses a larger region that the V6 sequences, the HF8 and Lachno2 primers were BLASTed against the complete reference sequences dataset that corresponded to the shorter V6 sequences. The V6 sequencing reads, each a proxy for a bacterial community member, were then binned within the HF8 cluster or Lachno2 cluster if their corresponding reference sequences contained both the forward and reverse primers and the probe sequences for the assays.

### Infection risk model

To examine the association between proximity to fecally contaminated water and schistosomiasis, a linear regression model was created with SPSS version 19 and ArcGIS 10.1. For the model, we made the following simplifying assumptions: 1) infection occurs at the common water contact sites, 2) probability of infection depends only on proximity of place of residence to a water contact site, 3) distribution of snails along the river is homogeneous, and 4) prevalence of snail infection is proportional to degree of human fecal contamination in water.

In order to associate spatial distribution and the prevalence of *S. mansoni*, the residential area of Jenipapo was mapped as a grid of 200 m^2^ blocks. The density of human population and density of number of cases of schistosomiasis per block was calculated using the point density tool in ArcGIS. The prevalence of schistosomiasis within each block was obtained by calculating the ratio of these two values using the Map Algebra tool. All houses within a 200 m^2^ block along the river were assigned the mean prevalence for that block. This spatial prevalence for each household then comprised the dependent variable in the linear regression. The independent variable was the risk of exposure to fecal contamination. To assign a value for fecal exposure for each household, spatial interpolation of two fecal marker DNA concentrations measured from the eight-water sample sites was performed using Inverse Distance Weighting (IDW). The village was divided into a two-dimensional grid of cells whose values were a function of their distance from a water contact site and the concentration of a fecal contamination marker at that site. A power of 2 was determined to be the best value for the weighting exponent by distance with a cell size of 20 m. Since human fecal contamination of water is necessary for transmission of *S. mansoni*, we hypothesized that a human-indicative fecal marker (Lachno2) would be a better predictor of schistosome infection than a general fecal marker, i.e. *E. coli*. Consequently, *E. coli* and Lachno2 estimated concentrations at each resident's location were extracted from the IDW generated surface to obtain an *E. coli*-IDW and Lachno2-IDW value for each home. Water samples were not taken from the Brejões River, thus, the section of the community bordering the Brejões was not included in the analysis. The relationship of fecal contamination to prevalence of schistosomiasis was then assessed by standard linear regression. The model significance was determined by bootstrapping with 1000 resamples at the household level.

## Results

### Study site and population

In 2009, Jenipapo consisted of 128 houses with 482 residents. Twenty-three residents had no house assigned, hence were not included in the analysis (Table 2). More than 98% of residents had tap water and an indoor flush toilet. There was access to adequate sanitation for 201 (43.8%) via home septic tanks, while sewage drained directly into the river for the remaining 258 (56.2%). Schistosomiasis was found in 209 individuals (45.5%) by examination of 3 stools collected on different days [22]. The geometric mean of intensity (57 epg) indicates that infections are generally light and comparable to other studies in Brazil [30]. Ten percent of the infections were heavy (>400 epg).

**Table 2:** Population characteristics.

Characteristics	
Population	459
Houses	128
Residents/house	3.6
Sex	
Male (%)	221 (48.1)
Mean Age (SD)	30.5 (21.6)
% Age <15	28
Tap water (%)	
Yes	459 (100)
Flush toilet (%)	
Yes	453 (98.7)
Sewage destination by house (%)	
Septic tank	68 (53.2)
River	60 (46.8)
*S. mansoni* infection	
Prevalence (%)	209 (45.5)
Mean intensity epg (S.D.)	57 (4.1)

### Spatial distribution of human population, sewage, and schistosomiasis

The Jiquiriçá River flows from west to east, and its course measures 1542 meters from the upstream sampling site to the downstream site. There is one formal bridge across the river at the point where the Brejões River enters the Jiquiriçá. Human water contact sites were primarily used to cross the river as well as for bathing, washing clothes, or fishing (Fig. 1a). The majority of houses are located on the south bank. Kernel density estimation shows clustering of human population density at both ends of the village along the south side of the Jiquiriçá River, but the greatest density clustered along the Brejões (Fig. 1b). The distribution of houses with sewage draining directly into the river (Fig. 1c), however, were not clustered based on Moran's I statistic, which fell within the 95% confidence interval of the null hypothesis (random spatial distribution) as indicated by a low z-score (Moran's index = −0.013, p = 0.847, z = −0.19). In contrast, for the prevalence of schistosomiasis, kernel density estimation shows clusters located along the Brejões River and for people living along the most downstream segment of the village (Fig. 1d). The positive Moran's index with a high z-score and low p value indicated that the distribution of schistosomiasis was not random (Moran's index = 0.042, p = 0.006, z = 2.74).

### Marker specificity

The 16S rRNA sequencing reads of extracted DNA from fecal samples of ten humans and all collected animal fecal samples were normalized against their maximum number of reads and queried for the human-indicative *Bacteroides* HF8 and Lachno2 clusters. Overall, humans had considerably lower amounts of *Bacteroides* in relation to *Lachnospiraceae* or more specifically, *Blautia* (one genera within *Lachnospiraceae* from which the Lachno2 assay was designed). Despite the low amount of overall *Bacteroides* in humans, the HF8 sequence represented 28% of all *Bacteroides* sequences. Overall, the proportion of sequence reads matching the HF8 cluster in humans was 10-fold higher than for pigs and dogs, and 100-fold higher than for horses and cows. The Lachno2 cluster showed even higher specificity with the proportion of reads in humans ∼100-fold higher than three animal sources, but ∼10-fold higher than for horses (Table 3).

**Table 3:** Percent of sequences matching HF8 and Lachno2 clusters by fecal source.

Average	Human	Cow	Pig	Dog	Horse
*Bacteroides* % of total	0.29	0.058	3.4	0.097	3.3
HF8 % of total	0.11	0.001	0.013	0.011	0.001
*Lachnospiraceae* % of total	17.70	0.071	12.5	0.14	7.5
*Blautia* % of total	1.2	0.006	0.014	1.3	0.33
Lachno2 % of total	0.081	0.001	0.001	0.002	0.009

The 16S rRNA gene was sequences from total DNA extracted from stool or filtered fecal sediment (Human). There were ∼1 million sequences per sample generated and matched to species or family. All fecal samples were collected from the village of Jenipapo, 10 humans, 2 cows, 3 pigs, 3 dogs, and 2 horses. Species of the genus Blautia are one component of the Lachno2 group.

### Spatial distribution and sources of fecal contamination in the river

**Figure 2:**
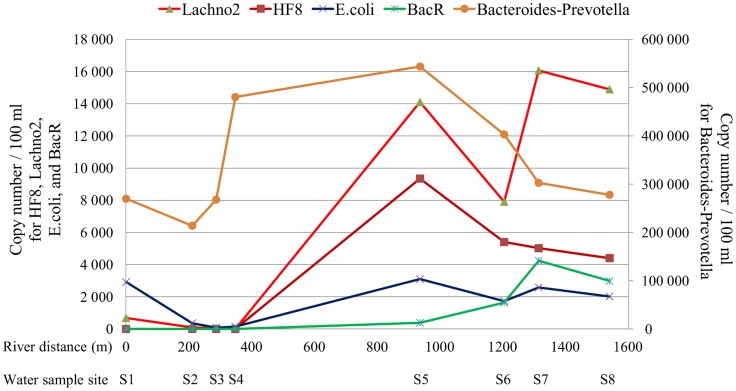
Distribution of bacterial concentration along the Jiquiriçá River. qPCR measurement of target DNA from anaerobic bacterial families and *E. coli* in water samples collected along the length of the river in Jenipapo, Bahia, Brazil. S1–S8 primary sites of human water contact. Distances along the river are provided in meters. *Bacteroides* and *Prevotella* - general, Lachno2 - human-indicative Lachnospiraceae gene cluster, HF8 - human-indicative Bacteroidales gene cluster, BacR - ruminant-indicative Bacteroidales gene cluster.

Using qPCR, the concentration of the *Bacteroides*-*Prevotella* group was at its lowest (<2.7×10^5^ copies/100 ml) from site S1, located upstream of the first house of the village, through site S3 (Fig. 2). There was a steep increase at S4 to 4.8×10^5^ copies/100 ml. The highest concentration was found at S5 (5.4×10^5^ copies/100 ml), where the Brejões River joins the Jiquiriçá. Its concentration then decreased gradually and by S8, located downstream of the last house, the concentration of this general fecal marker had returned to a value similar to S1 (2.7×10^5^).

**Figure 3:**
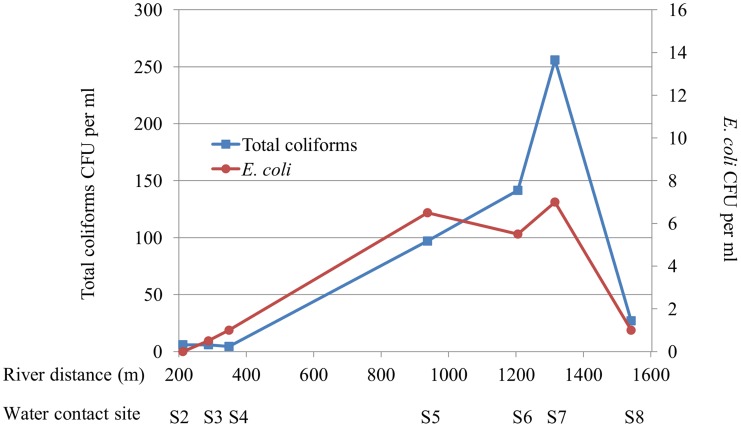
Distribution of *E. coli* and coliform concentration by bacterial culture along the Jiquiriçá River. S1–S8 primary sites of human water contact. Distances along the river are provided in meters. CFU - colony forming units. No sample was collected for site 1.

The human-indicative markers (HF8 and Lachno2) followed a similar distribution, however, concentrations increased one site further downstream compared to the *Bacteroides-Prevotella group* marker. The HF8 marker was undetectable until site S5, at which point it also reached its peak (0.9×10^4^ copies/100 ml), followed by a gradual decline. Lachno2 was detectable in minimal quantities at sites S1 to S4 (maximum concentration 684 copies/100 ml), and also had a marked increase by site S5. The peak Lachno2 concentration was at site S7 (1.6×10^4^ copies/100 ml), which is the last site downstream in Jenipapo that humans utilize to cross the river, and declined by S8. The ruminant-indicative marker was undetectable until S5 and remained in low concentrations without significant variation between sites thereafter. The *E. coli* marker showed a smaller degree of increase after S5. By contrast, the source of drinking water located 4.8 km north of the village had no copies of the HF8 human-indicative marker; other assays were not performed on this sample. Colony counts for coliforms, and less so for *E. coli*, also increased as the river moved down stream and declined sharply past the last house in the village (Fig. 3).

### Changes in bacterial communities across the river transect

**Figure 4:**
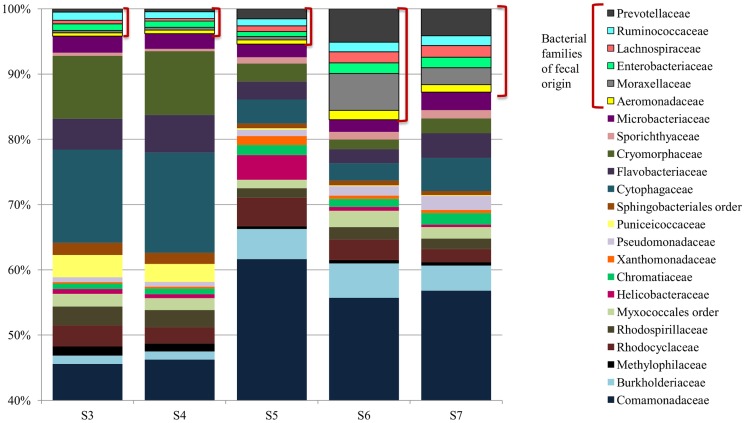
River microbial communities (Family level). River samples were collected from five points along the Jiquiriçá River in Jenipapo, Brazil on August 18, 2012. Microbial community populations of five river samples. The V6 hypervariable regions of the 16S rRNA gene from community genomic DNA were amplified and sequenced using Hi-Seq Illumina Sequencing. Taxonomy was assigned to sequences using GAST and taxonomic counts were normalized to the maximum number of sequences. Only the most abundant genera (>1% of at least one sample) are presented. Families discussed in the text are outlined.

Sequence data from the microbial communities found in river water was used to compare the relative abundance of >20 bacterial families. Consistent with the qPCR results, the proportion of *Prevotellaceae* and *Lachnospiraceae* increased significantly in the downstream portion of the village (Fig. 4). *Ruminococcaceae* and *Enterobacteriaceae*, two other families associated with fecal communities, also increased. These combined fecal families increased their representation from ∼3% to ∼9% of all bacterial community between upstream to downstream sites. Families associated with sewage- contaminated water - *Moraxellaceae* and *Aeromonadaceae*, specifically *Acinetobacter* spp. and *Aeromonas* spp. [31]–[34] - also increased at sites six and seven. *Comamonadaceae*, a bacteria common to the environment and freshwater [35], was the most abundant family on average, accounting for over 40% of the microbial community populations at each sampling site. *Bacteroidaceae*, which includes the genera *Bacteroides*, were in low abundance and are not represented in Figure 4.

### Site-specific risk model

**Figure 5:**
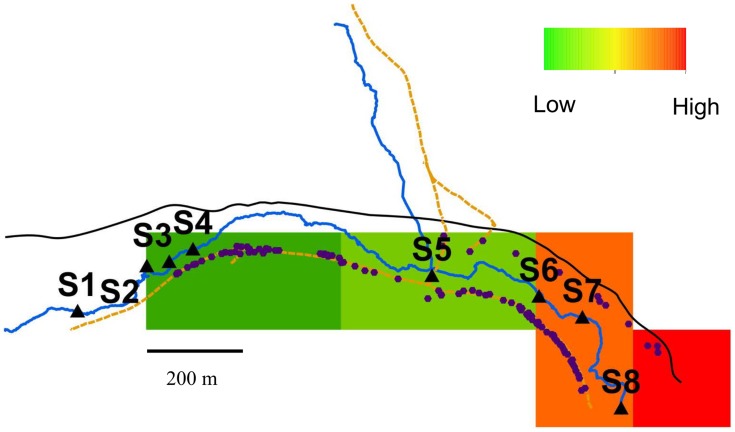
Map of Jenipapo with the area of houses divided in a 200 m^2^ grid. Schistosomiasis prevalence for each 200 m^2^ block was estimated. Infection prevalence increased downstream.

The 200 m^2^ schistosomiasis prevalence grid for Jenipapo produced 7 blocks (Fig. 5). Each house was assigned a value for exposure to fecal contamination based on proximity to a water sample site and the fecal marker concentration at site. The relationship of risk for infection with *S. mansoni* to the concentration and proximity to fecal contamination was modeled and tested statistically using the data from Jenipapo. Linear regression of prevalence of schistosome infection against fecal contamination yielded an r^2^ of 0.28 for the *E. coli*-IDW value (two-tailed p<0.001, 95% CI 0.22–0.35) and 0.53 for the Lachno2-IDW value (two-tailed p<0.001, 95% CI 0.48–0.58). These results can be interpreted as local concentration of human fecal contamination explaining over 50% of the variance in risk for schistosomiasis.

## Discussion

Although the village of Jenipapo is small, it is typical of many villages of Latin America. It also shares a pattern of development common with larger communities and even the great metropolises of Brazil. The village grew up along the two rivers that meet at its center, and most homes border these rivers in part to have access to a ready form of sewage removal. The community's drinking water supply is 4.8 km away where a dammed stream forms a small reservoir. Jenipapo's geometry is a simple, mostly linear distribution of residences and water contact sites, and this made it ideal for studying the dynamics of fecal contamination and its relationship to acquisition of schistosomiasis.

Putting the degree of fecal contamination of the Jiquiriçá River within Jenipapo in context, the geometric mean CFUs for *E. coli* (113 CFU/100 ml) was at the upper limit of the EPA's 2012 Recreational Water Quality Criteria value of 100 CFU/100 ml [2]. This level of contamination was estimated to result in 32 gastrointestinal illnesses per 1000 primary recreational contacts.

We were further able to identify human waste as the major contributor to this contamination. We validated both the HF8 and Lachno2 genetic markers as human-indicative by directly assaying the resident population. Interestingly, both human-indicative markers were identified from humans in the US, but were also significantly associated with humans in Brazil. The frequency of members of the *Prevotella* complex were higher in this rural Brazilian population than in communities in countries like the US and Italy where fat and protein form more of the diet than cereals, with Bacteroides a minor component of the human samples. In all human communities, including hunter gatherers, the *Lachnospriaceae* group, however, is more similarly represented [36]. In comparison with other human indicative markers, Lachno2 showed a high signal in the water sample and all human feces, but near absence in cows, the other major animal contributing to fecal contamination of the river. These markers indicated that human waste was the major contributor of fecal contamination in this section of river. Overall, human-indicative fecal indicators contribute important quantitative information on water quality that could be used for surveillance to gauge specific sanitation interventions.

The nearest community to Jenipapo is 8 km upstream with a population of 353 and similar level of sanitation, and there are few intervening houses, but many areas of pasture. Twelve km further upstream there is a town of 12000. Despite nearby populations, quantitative tracking of human fecal contamination in this study suggests a predominance of local effects. The qPCR markers for human and other fecal contamination, as well as coliform colony counts, are very low at the entrance to the village and significantly increase as the river continues downstream. Inflow for the village has significant levels of the *Bacteroides-Prevotella* group, but is very low for human fecal contamination indicating that most influence from communities upstream has dissipated. We presume this is not the result of the HF8 marker being sensitive to environmental degradation, since experimentally the duration of signals from *Bacteroides* ranges from days to several weeks [37]. In addition, the other marker of human fecal contamination (Lachno2) shows a similar pattern. Within Jenipapo, the entry of sewage is not clustered to one area of the community, and we noted the concentration of contamination is cumulative as the river moves downstream through the village.

The analysis of bacterial communities was based on number of sequence reads and is consistent with the qPCR genetic marker data. The study is limited in the relatively small number of samples taken, sampling only ∼50 m beyond the community's houses and a lack of household water samples. Also the human and animal samples were handled differently, but the distribution of bacterial families is consistent with other studies of the human gut biome [38]. A major strength of the study is the use of markers highly informative for the presence of human feces. Traditional indicators, such as E. coli culture and PCR, are able to demonstrate some of the same distribution pattern as HF8 and Lachno2, but the better model fit using Lachno2 demonstrates that higher tier assessments like qPCR for human indicative markers may provide better linkages between disease and human sources.

Human fecal contamination of water and the presence of snails are prerequisites for transmission of schistosomiasis. Snails are known to have a limited range of movement [39]. Proximity to water bodies where there are infected snails is a known risk factor for schistosomiasis [40], [41]. However, all inhabitants in Jenipapo are essentially equidistant from the river, and finding and determining which snails are infected can be laborious. In this study we show that, in a village with high prevalence of schistosomiasis, the risk of acquiring the infection is driven not only by proximity to surface water but also by its degree of human fecal contamination. The model explained a large amount of variation without including data on snail populations. We also observed that parasite populations were genetically more similar among infected members of the same household compared to parasite populations of all infected individuals in the village [21], which further supports the local, household level of acquisition. The variation not explained by our model was likely due to violations of our simplifying assumptions. Snails are not likely to be evenly distributed, and infection risk is influenced by more than distance to a contact site (age, type of activity, etc.). Some infection occurs outside of contact sites or not at the nearest contact site.

Although the human population disperses widely over this area, the local opportunities for exposure near the home may dominate the infection risk profile. Since awareness of schistosomiasis has been raised in the community and well before the analysis of fecal contamination, we have heard reports that teenage boys now prefer to enter the river upstream of the village. This may be a wise precaution, although the better solution will be to remove the contamination from the river rather than remove the boys and girls.
